# Effects of DHA- Rich n-3 Fatty Acid Supplementation on Gene Expression in Blood Mononuclear Leukocytes: The OmegAD Study

**DOI:** 10.1371/journal.pone.0035425

**Published:** 2012-04-24

**Authors:** Inger Vedin, Tommy Cederholm, Yvonne Freund-Levi, Hans Basun, Anita Garlind, Gerd Faxén Irving, Maria Eriksdotter-Jönhagen, Lars-Olof Wahlund, Ingrid Dahlman, Jan Palmblad

**Affiliations:** 1 Department of Medicine, Karolinska Institutet, Karolinska University Hospital, Huddinge, Stockholm, Sweden; 2 Department of Neurobiology, Caring Sciences and Society, Karolinska Institutet, Karolinska University Hospital, Huddinge, Stockholm, Sweden; 3 Department of Public Health and Caring Sciences, Uppsala University Hospital, Uppsala, Sweden; 4 Division of Geriatrics, Uppsala University Hospital, Uppsala, Sweden; 5 Division of Clinical Nutrition and Metabolism, Uppsala University Hospital, Uppsala, Sweden; South Texas Veterans Health Care System and University Health Science Center San Antonio, United States of America

## Abstract

**Background:**

Dietary fish oil, rich in n-3 fatty acids (n-3 FAs), e.g. docosahexaenoic acid (DHA) and eicosapentaenoic acid (EPA), regulate inflammatory reactions by various mechanisms, e.g. gene activation. However, the effects of long-term treatment with DHA and EPA in humans, using genome wide techniques, are poorly described. Hence, our aim was to determine the effects of 6 mo of dietary supplementation with an n-3 FA preparation rich in DHA on global gene expression in peripheral blood mononuclear cells.

**Methods and Findings:**

In the present study, blood samples were obtained from a subgroup of 16 patients originating from the randomized double-blind, placebo-controlled OmegAD study, where 174 Alzheimer disease (AD) patients received daily either 1.7 g of DHA and 0.6 g EPA or placebo for 6 months. In blood samples obtained from 11 patients receiving n-3 FA and five placebo, expressions of approximately 8000 genes were assessed by gene array. Significant changes were confirmed by real-time PCR. At 6 months, the n-3 FAs group displayed significant rises of DHA and EPA plasma concentrations, as well as up- and down-regulation of nine and ten genes, respectively, was noticed. Many of these genes are involved in inflammation regulation and neurodegeneration, e.g. *CD63, MAN2A1, CASP4, LOC399491, NAIP, and SORL1* and in ubiqutination processes, e.g. *ANAPC5 and UBE2V1*. Down-regulations of *ANAPC5* and *RHOB* correlated to increases of plasma DHA and EPA levels.

**Conclusions:**

We suggest that 6 months of dietary n-3 FA supplementation affected expression of genes that might influence inflammatory processes and could be of significance for AD.

**Trial Registration:**

ClinicalTrials.gov NCT00211159

## Introduction

Omega-3 fatty acids (n-3 FAs), e.g. eicosapentaenoic acid (EPA; 20∶5 n-3) and docosahexaenoic acid (DHA; 22∶6 n-3), present in marine oils, modulate inflammatory reactions and ameliorate symptoms of several autoimmune and other inflammatory disorders [1], [2]. In addition, EPA and DHA administration reduces cardiovascular morbidity and mortality, e.g. from ventricular arrhythmias [3]. Recently, high fish intake or dietary supplementation with n-3 fatty acids has been linked to reductions in the risk of developing Alzheimer's disease (AD) [4], [5], [6] and to delayed cognitive decline in patients with very mild AD [7].

N-3 FA are considered to exert the anti-inflammatory effects on several cellular levels, including surface receptor modulation, ion pumps, G-proteins, binding to transcription factors (e.g. nuclear transcription factor κB /NFκB/ and other signalling systems), as well as on gene activation [8], [9], [10]. Previous investigations on effects of DHA and/or EPA on gene expressions in animal studies and *in vitro* models have shown changes in a variety of genes, some of which are believed to be involved in inflammation and chronic neurodegenerative disorder. These gene expression studies have mostly been conducted after a short time exposure and on small sets of genes [11], [12], [13], [14], [15], [16], [17], [18]. However, a microarray study on the cerebral cortex of neonate baboon after 10–12 weeks on a DHA-enriched formula showed changes in approximately 1000 probesets/genes (but none more than 2-fold) [19]. In murine studies, 3 weeks of dietary supplementation of fish oil changed five genes more than 2-fold, and DHA enriched fish oil for approximately 2 months identified 329 and 356 dietary regulated transcripts from liver and hippocampus, respectively [20], [21]. There were no published studies of effects of long-term treatment with EPA and DHA in humans, using genome wide techniques, until recently [22].

Here, we present results of a clinical trial, the OmegAD study [7], where a product rich in DHA was given to patients with mild to moderate AD. The goal of the OmegAD study was, inter alia, to see if this n-3 preparation would reduce the cognitive deterioration. In the present study of the OmegAD trial, we used global transcriptome profiling to detect new genes responding to DHA-rich n-3 supplementation in isolated peripheral blood mononuclear cells (PBMCs). Preliminary results from this study has been presented previously [23].

## Materials and Methods

### Subjects

**Figure 1:**
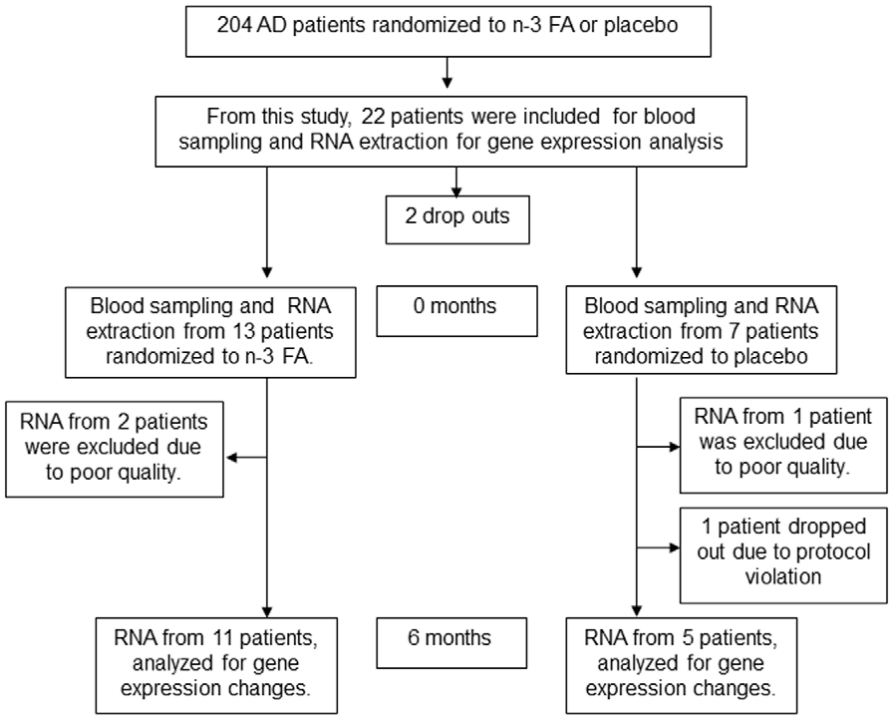
CONSORT flowchart. Study design at baseline and after n-3 FA or placebo supplementation to AD patients for 6 months. AD = Alzheimer's disease, n-3 FA = Omega-3 fatty acid treatment, Placebo = Placebo treatment.

This per-protocol study, included finally 16 patients (see Supplementary Material **S1**) for details of in- and exclusions), **Figure 1**. They were among the first to be randomized in the OmegAD study, described in detail in [7]. In summary, the double blind, placebo controlled OmegAD study included 204 patients (73±9 y, 52% women) with mild to moderate AD. Patients were randomized to 6 months of nutritional supplementation with a marine n-3 fish oil rich in DHA or to placebo. Patients were treated daily with either 1.7 g DHA plus 0.6 g EPA (EPAX 1050TG; Pronova Biocare A/S, Lysaker, Norway) or with isocaloric placebo oil (1 g corn oil, including 0.6 g linoleic acid) for 6 months. EPAX 1050TG is a 60% n-3 fatty acid concentrate in triacylglycerol form, produced according to good manufacturing practices. Four milligrams of vitamin E (tocopherol) was added to each EPAX 1050TG and placebo capsule. A total of 174 patients completed the OmegAD study. Plasma fatty acid profiles, cognition and behavioural data have been published [7], [24]. A pre-trial power calculation in the whole study, estimated that 200 patients have to be included with a statistical significance level of 0.05 and 80% power to accomplish differences in the measurement of the cognitive function. The estimation of numbers of enrolled patients in our gene expression subgroup by power calculation has not been applicable, due to insufficient knowledge as to variables included in the power calculation for the thousands of genes measured by the microarray technique. Here, we present data on 11 (54–82 y; median 71, and 5 woman) patients, who received the n-3 FAs preparation, and 5 patients (56–80 y; median 66, and 2 woman), receiving the placebo oil, for 6 months.

No changes in peripheral blood neutrophil, monocyte, and lymphocyte cell counts were recorded after 6 months of n-3 FAs treatment. In this study group, no specific advice on food intake was given, since included patients suffered from AD.

### Blood sampling

EDTA anticoagulated venous blood was obtained prior to or after 6 months of n-3 FA treatment. PBMCs were isolated by means of a Lymphoprep (Nycomed Pharma, Oslo, Norway) gradient centrifugation. The cell preparations obtained before and after treatment with n-3 FAs, contained on average 14% monocytes and 86% lymphocytes on both occasions. Corresponding figures for the placebo group were 15% and 85%, respectively. The cell viability in both groups was on average 96%, as assessed by trypan blue staining.

### RNA extraction and microarray hybridization

Total RNA was isolated from PBMCs according to protocols given in Supplementary Material **S1**. From non-degraded high-quality total RNA we prepared biotinylated complementary RNA that served as template for hybridization to Human Genome Focus Arrays (Affymetrix, Inc., Santa Clara, CA) containing 8747 probesets, corresponding to approximately 8000 genes (see Supplementary Material **S1**).

### Microarray data analysis

Information about calculation of signal intensities for each microarray and quality control in Microarray Analysis Suite (MAS) 5.0 (Affymetrix) are given in Supplementary Material **S1**. Genes were categorized using the Gene Ontology (GO) database.

We used the software Significance Analysis of Microarrays (SAM) [25] to identify changes in gene expression induced by n-3 FA treatment. Details of this procedure are given in Supplementary Material **S1**). In addition, we used SAM for sample size calculation. Our dataset had limited power to detect differentially expressed genes. However, the purpose of the study was not to characterize the complete response of gene expression to the intervention, but to detect a few responding genes.

### Verification of microarray with real time PCR

We selected genes that were interesting biological candidates for PCR confirmation, with the restriction that this selection should not be biased and only represent those with the largest fold change. RNA was reversed transcribed into cDNA, using an iScript cDNA synthesizing kit. Real time-(RT)- PCR was performed in an iCycler IQ (Bio-Rad Laboratories Inc., Hercules, CA). The results were normalized to low density lipoprotein receptor-related protein 10 (LRP10), whose mRNA levels were unaffected by n-3 FA and placebo treatment. Details are given in Supplementary Material **S1**.

### Plasma fatty acid analyses

Plasma fatty acids (FAs) were analyzed by gas chromatography (Thermo TR-Fame column (30 m×0.32 mm ID×0.25 µm film; Thermo Electron Corp, Waltham, MA) and are given as relative abundance of individual fatty acids [26] in each participating individual. Data for all 174 patients in the OmegAD study have been given previously [7]. Changes of the EPA and DHA plasma concentrations are given as the difference of percentage units.

### Statistical analyses

Paired two sided Students' t-tests were used to analyze changes in plasma FAs- and gene expression data. For microarray data, the results for the n-3 FA group were adjusted for multiple comparisons using SAM (see above). A two-sided independent Students' t-test was used to compare fold change between the n-3 FA and placebo groups. Paired one-sided Students' t-tests were used in confirmatory analysis of individual mRNA measurements by RT-PCR. Values are presented as mean±SD. For correlation analyses, Pearson correlation tests were applied. *P* values<0.05 were considered significant.

## Results

### Plasma fatty acids

**Figure 2:**
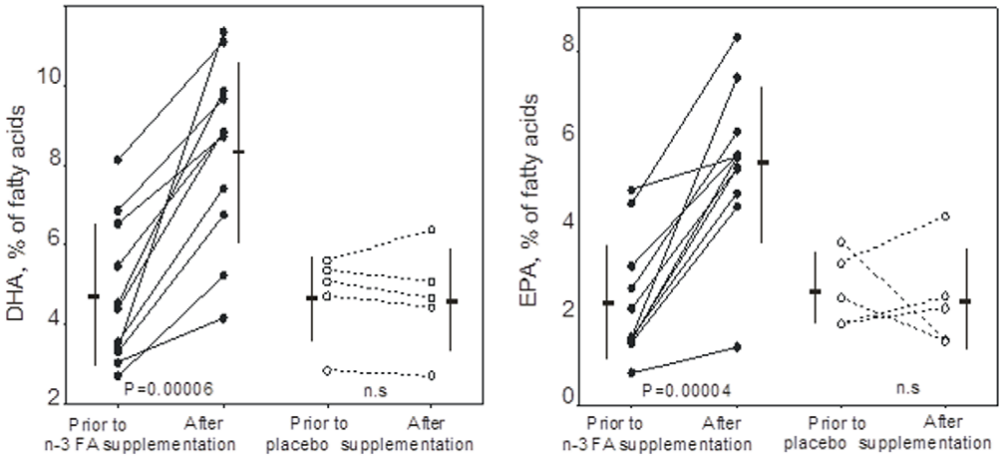
Plasma values for docosahexaenoic acid (DHA) and eicosapentaenoic acid (EPA) at baseline and after 6 months. Individual values are flanked by mean and SD values. Significant increases of DHA and EPA were noted in the n-3 fatty acids (n-3 FAs) group after 6 months, but not in the placebo group.

At study entry, plasma concentrations of EPA and DHA did not differ significantly between the two groups (**Figure 2**). In the n-3 FAs group plasma values for DHA as well as for EPA were significantly higher at 6 months compared with pre-trial values (p = 0.00006 and p = 0.00004 respectively). The placebo group displayed no significant alterations of DHA and EPA in plasma compared with pre-trial values (**Figure 2**). The mean rise of DHA levels was slightly larger than that of EPA in the n-3 FA group (+3.6 percentage units and +3.14 percentage units, respectively). Nonetheless, here, and as in our previous reports [7], [24], EPA values were enhanced more than expected from the ratio of orally supplemented EPA to DHA, being 0.35∶1.

### Gene expression by microarray

Next, we assessed which genes, out of the 8000 on the microarrays that showed a significant change in expression in PBMCs following n-3 FA supplementation. All microarrays fulfilled the employed quality criteria (see Supplementary Material **S1** for details). On the microarrays 4,075 probesets gave signals that, according to the criteria in MAS 5.0, showed that the corresponding mRNA was expressed in PBMCs on >15 of 22 arrays in the intervention group. Such genes were scored as present in PBMCs and were further analyzed in SAM. According to SAM analysis the expression of 19 genes were up- or down-regulated in PBMCs by 6 month treatment with n-3 FA with a false discovery rate of 10%.

### Gene changes in the n-3 FA group

**Table 1** presents results for significantly up-regulated genes in PBMCs, after n-3 FAs supplementation for 6 months. The nine up-regulated genes in the n-3 group are presented below, in falling order of magnitude, and with comments on possible functions (partly provided by Ref Seq at: http://www.ncbi.nlm.nih.gov/RefSeq/).

**Table 1:** Genes up-regulated by a DHA rich n-3 fatty acid or placebo supplementation.

		n-3 FA group^1^		Placebo group
*Locus Link*	*Genes*	*Fold change*		*Fold change*
*MS4A3*	Membrane-spanning-4 domains subfamily A	1.72±0.83	0.0037	1.65±1.12
*NAIP*	NLR family, apoptosis inhibitory protein	1.37±0.35	0.0028	1.16±0.11
*DRG1*	Developmentally regulated GTP binding protein 1	1.20±0.19	0.0033	1.14± 0.11^2^
*CD63*	CD63 molecule	1.20±0.18	0.0019	0.99±0.11^3^
*HSD17B11*	Hydroxysteroid (17-beta) dehydrogenase 11	1.19±0.15	0.0006	1.27±0.55
*RAB27A*	Member RAS oncogen family	1.18±0.16	0.0024	1.08±0.18
*CASP4*	Apoptosis-related cysteine peptidase	1.17±0.14	0.0026	0.99±0.20^4^
*SUPT4H1*	Suppressor of Ty 4 homolog 1	1.15±0.11	0.0008	1.02±0.12^4^
*UBE2V1*	Ubiqutin-conjugating enzyme E2 variant1	1.13±0.10	0.0012	1.06±0.22

All values are means±SD.

^*1*^All genes were significantly up-regulated by the n-3 FAs treatment according to SAM based on 2000 permutations and a false discovery rate of 10%, see microarray data analysis for details.

^*2*^We analyzed by t-test if genes regulated by n-3 FAs supplementation were also up- or down-regulated in the placebo group. The change for *DRG1* was significant (p = 0.04).

^*3*^Significant and

^*4*^close to significance (p = 0.05–0.06) difference in fold change between the n-3 FAs and placebo group.

*MS4A3* (also known as *CD20L and HTM4*) encodes for a member of the membrane-spanning 4A gene family. The corresponding protein likely plays a role in signal transduction and may function as a subunit associated with receptor complexes.

*NAIP* (also known as *BIRC1*) codes for a protein belonging to the NLR family of apoptosis inhibitory proteins (AIP). It is thought that this gene is a modifier of spinal muscular atrophy caused by mutations in a neighbouring *SMN1* gene. A low level of *NAIP* protein has been found in AD brains. Further, it has been suggested that a rise of the *NAIP* protein level protects AD patients against the development of tangle pathology and cognitive decline [27]. Thus, changes in this gene might be of significance for the population of AD patients studied here.

*DRG1* (also known as *NEDD3, PROXY1, and NDRG1*) encodes for the developmentally regulated GTP binding protein-1. This gene is a member of the N-myc down-regulated gene family, which belongs to the alpha/beta hydrolase superfamily. The encoded protein is cytoplasmic, involved in stress and hormone responses, cell growth, and differentiation. It is necessary for p53-mediated caspase activation and apoptosis. Mutation in this gene has been reported to be causative for a hereditary variant of Charcot-Marie-Tooth disease (type Lom), and is, thus, linked to a degenerative nerve disorder.

The *CD63* (also known as *LAMP-3, OMA81H, and TSPAN30*, granulophysin) gene encodes for the CD63 antigen (also known as melanoma-1 antigen) found e.g. in tissue macrophages. This cell-surface protein belongs to the family of tetraspanin proteins involved in cell adhesion and cell migration as well as neutrophil granule mobilization [28]. Thus, it is clearly involved in regulation of inflammation.

The enzyme encoded by *HSD17B11*, hydroxysteroid (17β) dehydrogenase-1 (also known *as DHRS8*), modulates the intracellular glucocortocoid levels and takes part in the steroid and lipid biosynthetic processes. Again, this protein is involved in regulation of inflammation.

*RAB27A* (also known as *RAM, RAS-associated protein RAB27A*). The protein encoded by this gene belongs to the small GTPase superfamily, Rab family. The protein is membrane-bound and may be involved in protein transport and small GTPase mediated signal transduction. Mutations in this gene are associated with Griscelli syndrome type 2, an X-linked lymphoproliferative syndrome associated with hemophagocytic syndromes [29], indicating that this protein is involved in regulation of inflammation.

*CASP4* (also known *as ICE (rel) II*, *ICEREL-II*, and *ICH-2*) codes for caspase-4, an apoptosis-related cysteine protease, classified as one of the inflammatory caspases. It participates in the LPS induced TLR4-signaling pathway, acting as an essential protein in the NF-κB activation of cytokine and chemokine production. Hence, it is an important part of the innate immunity system [30]. Moreover, caspase-4 is considered as a key caspase in apoptotic signalling in AD [31].

*SUPT4H1* (also known as Suppressor of Ty4 homolog 1). The protein from this gene has transcription factor activity and is involved in RNA synthesis.

The ubiquitin-conjugating E2 enzyme variant-1 protein (encoded by the gene *UBE2V1*, also known as *CROC1* and *UEV1A*) is located in the cell nucleus and can cause transcriptional activation of the human FOS proto-oncogene. This protein belongs to the ubiquitin-conjugating enzyme family but has no ligase activity on its own but is one of the enzymes involved in the ubiquitination of proteins.

As shown in **Table 2**, we found a **decreased expression** of 10 genes after 6 months of n-3 FAs treatment. They were, in falling order of magnitude, in the n-3 group as follows.

**Table 2:** Genes down-regulated by a DHA rich n-3 fatty acid or placebo supplementation.

		n-3 FA group^1^		Placebo group
*Locus Link*	*Genes*	*Fold change*	*P-value*	*Fold change*
*RHOB*	Ras homolog gene family, member B	0.70±0.26	0.0037	1.30±0.79^3^
*VCP*	Valosin-containing protein	0.72±0.24	0.0047	0.94±0.16^4^
*LOC399491*	LOC 399491 protein	0.73±0.19	0.0032	1.09±0.32^3^
*ZNF24*	Zinc finger protein 24	0.76±0.18	0.000019	0.99±0.12^3^
*SORL1*	Sortilin-related receptor L (DLR class) A	0.77±0.21	0.0046	0.95±0.18
*MAN2A1*	Mannosidase alpha, class 2A, member 1	0.78±0.21	0.0029	1.01±0.19^4^
*PARP1*	Poly (ADP-ribose) polymerase family, member 1	0.80±0.16	0.0024	1.01±0.29^4^
*SSRP1*	Structure specific recognition protein 1	0.82±0.12	0.0022	0.98±0.23^4^
*ARIH1*	Ariadne homolog, ubiquitin-conjugating enzyme E2 binding protein	0.83±0.16	0.0027	0.87±0.16
*ANAPC5*	Anaphase promoting complex subunit 5	0.87±0.09	0.0008	1.04±0.17^3^

All values are means±SD.

^*1*^All genes were significantly down-regulated by the n-3 FAs treatment according to SAM based on 2000 permutations and a false discovery rate of 10%, see microarray data analysis for details.

^*3*^Significant and

^*4*^close to significance (p = 0.06–0.09) difference in fold change between the n-3 FAs and placebo groups.

*RHOB* (also known as *ARH6*) is a Ras homolog gene family member B gene that encodes for a Rho-related GTP-binding protein with GTPase activity, RhoB precursor protein. It is involved in cell differentiation and cell adhesion. In different cell models, RhoB can activate NF-κB signalling by modification of the RelA/p65 transactivation domain [32], implying a role in inflammation. Further, small GTPase RhoB has a critical role in vascular development.

Valosin containing protein (*VCP, also* known as *p97*) is a structural endoplasmatic reticulum protein associated with clathrin and heat shock protein Hsc70. It belongs to the family of putative ATP-binding proteins involved in vesicle transport and fusion and ubiqutin-dependent protein degradation. Polymorphism in this gene may be linked to late onset AD [33].

*LOC399491* encodes for a hypothetical protein, possibly a membrane protein and a protein in the neuropeptide signalling pathway. No pathology has so far been associated with this gene.

The zinc finger protein 24 gene (*ZNF24*, also known as *ZNF191* and *KOX17*) is related to *ZNF191*, which probably is associated with some hereditary diseases and cancers. *ZNF24* may function as a transcription factor.

*SORL1* (*also* known *as SORLA and LR11*) encodes for the sortilin-related receptor L (DLR class) A. The gene encodes for a protein that belongs to the family of vacuolar protein sorting 10 (VPS10) domain-containing receptor proteins, which is mainly found intracellularly in the paranuclear compartment and is strongly expressed in the central nervous system. It regulates processing of amyloid precursor protein (APP) in AD [34], and the expression of *SORL1* is reduced in the brain of AD patients, thus being of considerable interest for understanding of the AD pathology [35].

The gene Mannosidase α, class 2A, member 1 (*MAN2A1* also known as *MANA2*) encodes for a α-mannosidase-2 protein, which is a family member of the glycosyl hydrolases in the Golgi apparatus. A mutation in a mouse homolog of this gene has been linked to a systemic autoimmune disease making it likely that this gene has a relation to inflammation regulation.

*PARP1* (also known as *ADPRT1*, *PARP-1*, *PPOL*, *pADPRT-1*). This gene encodes a chromatin-associated enzyme, poly (ADP-ribosyl) transferase-1, which modifies nuclear proteins. It is involved in the regulation of e.g. differentiation, proliferation, tumour transformation and the recovery of cells from DNA damage. This enzyme may be the site of mutation in Fanconi anaemia and may participate in the pathophysiology of type I diabetes. It has also been suggested that PARP-1 is involved in NF-κB driven expression of inflammatory mediators, like interleukin-1 (IL-1) [36]. An over activity of PARP-1 has been shown in the AD brain [37]. This gene is of interest for understanding of the AD pathology as well as inflammation.

The structure specific recognition protein-1 (*SSRP1*, also known as *FACT*, *FACT80* and *T160*). The encoded protein forms with other proteins the chromatin transcriptional elongation factor FACT, which appears to be crucial to the anticancer mechanism of cisplatin. This protein also functions as a co-activator of the transcriptional activator p63.

Ariadne homolog, ubiquitin-conjugating enzyme E2 binding protein-1 (*ARIH1*, also known as *ARI* and *UBCH7BP*), is one of the proteins involved in the ubiquitination process before degradation of proteins by the 26S proteasome.

The anaphase promoting complex subunit-5 protein (*ANAPC5* also known as *APC5*) gene encodes for the anaphase promoting complex/cyclosome (APC/C), a large E3 ubiquitin ligase that controls cell cycle progression by targeting a number of cell cycle regulators such as B-type cyclins for 26S proteasome-mediated degradation.

### Gene changes in the placebo group and comparisons with the n-3 FA group

We analyzed by t-test if genes regulated by n-3 FA supplementation were also up-or down-regulated in the placebo group. Only the *DRG1*gene were found to be significantly up regulated in the placebo group (p = 0.04; **Table 1**).

Eighteen of 19 genes, significantly changed by the n-3 FA supplementation, displayed a larger fold change in the n-3 FA group than in the placebo group; the exception was *HSD17B11* (**Table 1** **and** **2**). The difference was significant for five genes (*CD63*, *RHOB*, *LOC399491*, *ZNF24*, *ANAPC5*) and close to significance for an additional six (p = 0.05–0.09) (*MAN2A1*, *CASP4*, *SUPT4H1*, *VCP*, *PARP1*, *SSRP1*) by means of t-tests.

### Individual mRNA measurements

We chose five genes for validation by RT-PCR. Out of these five genes, two, *viz*. *CD63* and *SORL1* were significantly modulated in accordance with changes in the microarray assay, and *CASP4* was borderline significant (p = 0.05) (**Table 3**). The inability to confirm some genes could be due to false positive, i.e. the use of a 10% FDR, or limited power of the confirmation cohort.

**Table 3:** Individual messenger RNA measurements in the n-3 fatty acid group.

Locus Link	Accession no	Fold change^1^	*P*-value
*CD63*	Hs00156390_m1	1.18±0.25	0.02
*CASP4*	Hs00233437_m1	1.17±0.31	0.05^4^
*SORL1*	Hs00268342_m1	0.84±0.27	0.04
*RHOB*	Hs00269660_s1	0.84±0.41	0.11
*VCP*	Hs00200205_m1	0.97±0.14	0.22

^*1*^The expression of the target gene was normalized to the LRP10 internal control using the formula 2^(Ct target calibrator – Ct target sample)^/2^(Ct LRP10 calibrator – Ct LRP10 sample)^ where calibrator is a random sample. Subsequently, fold change was calculated as the ratio between values for 0 and 6 months of n-3 supplementation. Mean±SD values (n = 11).

^*4*^Near significance.

### Correlation analyses

**Figure 3:**
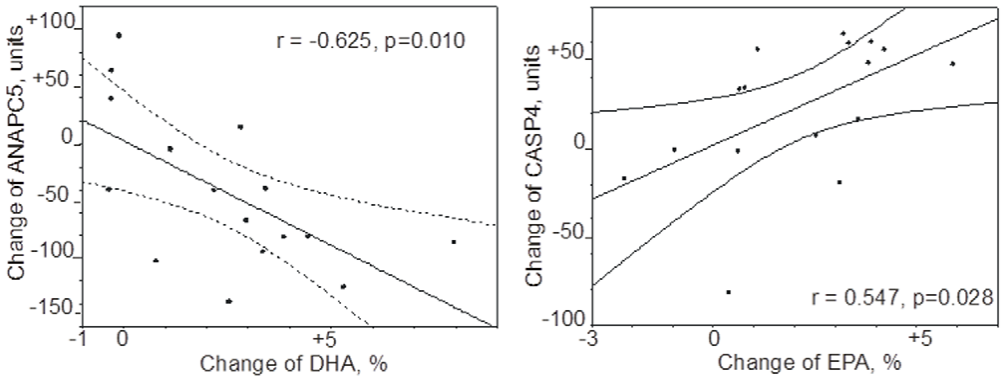
Correlation between changes of plasma docosahexaenoic acid (DHA) and eicosapentaenoic acid (EPA) and gene expression. Changes of plasma DHA, left, and EPA, right, and changes of expression (signal log ratio units) for the *ANAPC5* (left) and *CASP4* (right) genes.

When relating values for expressed genes to plasma concentrations of DHA or EPA, we found that changes in DHA or EPA for all 16 subjects correlated significantly to changes in the expression of *ANAPC5* (r = −0.625; p = 0.01 /**Figure 3** **left**/, and r = −0.619; p = 0.01, respectively), and *RHOB* (r = −0.512; p = 0.04, and r = −0.57; p = 0.02, respectively). Thus, the more DHA (or EPA) concentrations increased, the lower was the expression of the *ANAPC5* and *RHOB* genes. Moreover, we found that changes of EPA levels, but not DHA, for all 16 subjects correlated significantly to changes of the expression of the *CASP4* gene (r = 0.547; p = 0.028 /**Figure 3** **right**/). Thus, the *CASP4* gene expression rose with an enhanced EPA plasma level.

## Discussion

Here, we describe that 6 months of dietary supplementation of a DHA rich fish oil formulation to elderly humans afflicted with AD conferred significant up- or down-regulation of several genes. They represent a wide variety of cellular functions. Notably, many of these are associated with inflammatory reactions (*viz*. *CD63*, *HSD17B11*, *RAB27A*, *CASP4*, *MAN2A1* and *PARP1*), others with neuroinflammatory disorders (*viz*. *NAIP*, *DRG1*, *SORL1*, *CASP4* and *PARP1*), two processes which are highly relevant for actions of n-3 FAs and the aim of the OmegAD study to investigate. Some of the genes were found in both categories, emphasizing the inflammatory component of AD process [31], [38]. Moreover, only 1 (of 19) genes was significantly changed in the placebo treated group. Finally, the statistically significant relation between changes of plasma DHA and EPA levels and of the *RHOB* and *ANAPC5* genes is intriguing.

The four previously mentioned genome wide expression studies in baboons, rodents and the one in healthy subjects [19], [20], [21], [22] noted changes in 5–1000 genes in brains, livers and PBMC, respectively, after dietary DHA or EPA rich fish oil interventions for 3–10 weeks in rodents and 26 weeks in humans. However and surprisingly, none of those genes coincided with what we describe here as significantly up- or down-regulated. A possible explanation of these inconsistencies in results might be related to species, human populations and ages, FA types, doses, duration as well as to target organs. Thus, our study presents unique data on genes of relevance to long-term dietary supplementation with a DHA-rich preparation to aged and AD-afflicted humans.

Array techniques with a restricted set of gene probes (app. 3000 genes or less) were used in a few n-3 FA animal studies. In one, on brain tissue, the fish oil group displayed almost the same expression profile as the control group [11]. Another study found an up-regulation of the transthyretin gene in the hippocampus [12], whereas we did not in our study on human PBMC. In a third murine study, genes encoding for IL-1α, IL-1β or NO synthase were unaltered [13], as in our study. Two months of a DHA rich fish oil supplementation modified 77 of 588 studied genes in human lymphocytes [39] but, again, none was genes we identified as up-or down-regulated.

The recently published study on healthy humans given an EPA rich fish oil [22] (with total n-3 FA doses close to ours and for a similar time) observed changes in more than 1000 genes, where the magnitude of changes was often very small. The reason for the outfall in terms of number of genes can probably be related to using an array covering 17 000 genes, including approximately twice as many subjects as in our study, and using a nominal p-value 0.05 as threshold to determine significantly altered gene expression on microarray data. The latter makes the results difficult to compare with our analysis of microarray data with SAM, which takes multiple comparisons into account. Since only a fraction of all changed genes were listed in [22], we do not know if changes were seen in the same genes as in our study. Of note, out of 99 independent transcripts (changed by EPA) that could be identified in [22], 16 transcripts were not represented with probesets on our Focus array. Another 19 transcripts were detected in less than 29 of 32 arrays, and displayed very low signals; it has previously been demonstrated that variability in gene expression is a function of absolute expression, i.e. a lowly expressed gene is more variable than a highly expressed gene [40]. Results for such genes have an increased risk of being false positive and are also more difficult to confirm in independent datasets. In addition, some of the remaining 54 EPA-regulated transcripts in [22] gave low signals on our Focus array. Together, these factors can partially explain why we could not confirm the findings by Bouwens et al [22].

Several studies have examined the effects of fish oils on individual human genes and corresponding protein production, documenting, inter alia, diminished ex vivo pro-inflammatory cytokine production in mononuclear blood cells, e.g. TNF-α and IL-1β [41], [42] and IL-6 [42] and decreased TNF-α and IL-6 in a dose-dependent manner [43]. Fish oil also lowered gene expression of TNF-α in renal allograft after 3 months of treatment [44]. These findings could, however, not consistently be repeated in other studies [45], [46]. Again, the reason for these discrepancies may relate to n-3 FA dosage, treatment time, whether DHA or EPA was the predominant FA (since they may regulate genes differently) [17], [18], the cell type and the experimental design. This is further illustrated by rather heterogeneous results from animal studies [11], [12], [13] where species differences and age effects also are of significance.

As shown here, EPA as well as DHA plasma levels was enhanced in a similar way, suggesting that DHA was converted to EPA to some extent or that DHA was specifically cleared from plasma. This makes it difficult to attribute gene changes to one FA. However, DHA enriched formulas were rarely used in previous *in vivo* studies.

In a previous publication from the OmegAD study [24], we reported about changes of pro-inflammatory cytokine and growth factor production from LPS stimulated blood mononuclear cells ex vivo. However, we are unable to show any changes in corresponding genes. This might be due to our gene expression data is based on quiescent PBMCs, without LPS activation.

*In vitro* and after dietary supplementation, DHA increased the sortilin-1 receptor (*SORL1*) mRNA and protein in murine cortical neurons [47], but decreases have also been observed [48]. *SORL1* regulates the amyloid precursor protein and this protein is also elevated in aged DHA depleted mice [47]. In our study we found that n-3 FA down-regulated the expression of the *SORL1* gene which is in agreement with Perez et al [48].

Our DHA enriched n-3 FAs supplementation for 6 months caused effects with higher magnitudes on several genes compared with placebo treatment. Moreover, the genes *RHOB* and *ANAPC5* also showed a statistically significant correlation with the changes of both plasma DHA and EPA levels, making them interesting candidates for further studies on effects of n-3 FAs.

The magnitude of the gene expression changes observed here might appear rather small, since they ranged between +72 and – 30 per cent. This is, however, what might be expected in dietary supplementation studies in animals and humans [19], [22], [49]. In contrast, *in vitro* studies or in short-term animal studies larger changes might be found. One can anticipate that acute and large effects will vane with prolonged exposure because of an adaptation over time.

The present study gives novel information on mechanisms for marine lipids, suggesting that dietary n-3 FA supplementation affected expression of genes that might influence inflammatory processes and could be of significance for Alzheimer's disease. Recently, the significance of n-3 FA blood levels for telomeric aging in patients with coronary heart disease was demonstrated [50].

## Supporting Information

Supplementary Material S1 For details of patient in- and exclusion in the study, RNA extraction and microarray hybridization, microarray data analysis and verification of microarray with real time PCR.
(DOC)
