# Supplementary material for: Effects of DHA- Rich n-3 Fatty Acid Supplementation on Gene Expression in Blood Mononuclear Leukocytes: The OmegAD Study
Source: PLoS One. 2012 Apr 24;7(4):e35425. doi: 10.1371/journal.pone.0035425 (PMC3335851; doi:10.1371/journal.pone.0035425)
Supplement: Supplementary Material S1 — For details of patient in- and exclusion in the study, RNA extraction and microarray hybridization, microarray data analysis and verification of microarray with real time PCR. (DOC) [file pone.0035425.s001.doc]

SUPPLEMENTARY MATERIAL S1

Patient in- and exclusion

Because of logistic reasons 22 subjects donated blood for this study, for preparation of PBMCs or plasma. These 22 were among the first to be randomized into the trial; however, because of logistical reasons, they were not consecutive. Thus, the numbers of n-3 FA and placebo-treated patients became unequal. No other selections of patients were made. Subsequently, two of the patients dropped out of the trial. Out of the remaining 20 patients 13 had been randomized to the n-3 FA supplementation and 7 to placebo. However, some more patients and data were removed because of technical faults; these are given in Figure 1 (poor RNA quality, protocol violation). This study was evaluated on a per protocol basis.

RNA extraction and microarray hybridization

Total RNA was isolated from PBMCs according to the RNA purification RNeasy Mini Kit protocol and treated with RNase-free DNase (QIAGEN GmbH 40724 Hilden, Germany). RNA concentrations as well as purity (A260/A280 ratio) was measured spectrophotometrically using an Unicam UV-Visible spectrometry and by using Nanodrop ND-1000 Spectrophotometer (Thermo Fisher Scientific), and intact high quality, total RNA was confirmed using the Agilent 2100 Bioanalyzer (Agilent Technologies, Palo Alto, CA).

From this high-quality total RNA we prepared biotinylated complementary RNA that served as template for hybridization to Human Genome Focus Arrays containing 8747 probesets, corresponding to approximately 8000 genes. Labeling, hybridization, and scanning were prepared according to standard protocols from Affymetrix (Affymetrix Inc., Santa Clara, CA). After hybridization and scanning of the 32 arrays, each sample was analyzed using Affymetrix statistical algorithms.

Microarray data analysis

Signal intensities for each microarray were calculated in Microarray Analysis Suite (MAS) 5.0 (Affymetrix) with default parameter settings for the statistical algorithm. Using the all-probeset option, average intensities for each microarray sample was scaled to a target signal of 100, enabling multiple assay comparisons. Distinct algorithms were used to evaluate (i.e. present or absent) absolute calls for each transcript. Quality control was performed and Scaling factor and Number of present were well within approved intervals. Boxplot and Multidimensional Scaling showed an even distribution of signals and none of the samples represented an outlier, according to Affymetrix software GCOS and MAS 5.0.

We used the software Significance Analysis of Microarrays (SAM) (25) to identify changes in gene expression induced by n-3 FA treatment. SAM takes into account the multiple comparisons caused by the presence of thousands of probesets on the microarrays. The numerical analysis in SAM was based on paired comparisons (after vs before n-3 FAs treatment) of probesets with signal values scored as present on > 15 of 22 arrays in the intervention group according to the criteria in MAS 5.0. The analysis was based on 2000 permutations and a false discovery rate of 10%. Applying the SAM excel-macro to our data we could not get a higher resolution than either a 0 or a 10% FDR. We chose the 10% FDR since the 0% was considered too strict. Our Microarray data have been submitted to GEO in a MIAME-compliant format (GSE29680).

Verification of microarray with real time PCR.

300 ng of RNA was reversed transcribed into cDNA, using an iScript cDNA synthesizing kit (Bio-Rad Laboratories Inc, Hercules, CA). In a final volume of 20 µl, 2 ng of cDNA was mixed with TaqMan R universal mix (Applied Biosystems, by Roche Molecular Systems Inc, New Jersey, and USA) and pre-designed TaqMan gene expression assays from Applied Biosystems. Real time-(RT)-PCR was performed in an iCycler IQ (Bio-Rad). The thermal cycle program was as follows: 2 min at 50 º C and 10 min at 95 º C, followed by amplification of the cDNA for 50 cycles with melting for 15 seconds at 95 º C. Samples were run in duplicates. The results were normalized to low density lipoprotein receptor-

related protein 10 (LRP10, Hs00204094_m1, Applied Biosystems), whose mRNA levels were unaffected by n-3 FA and placebo treatment. The expression of the target gene was normalized to the LRP10 internal control using the formula 2(Ct target calibrator – Ct target sample)/ 2^(Ct LRP10 calibrator – Ct LRP10 sample)^ where calibrator is a random sample.
